# Deep learning for the screening of primary ciliary dyskinesia based on cranial computed tomography

**DOI:** 10.3389/fphys.2023.1098893

**Published:** 2023-03-15

**Authors:** Bo Duan, Hui-Ying Lv, Yue Huang, Zheng-Min Xu, Wen-Xia Chen

**Affiliations:** Department of Otolaryngology-Head and Neck Surgery, Children’s Hospital of Fudan University, Shanghai, China

**Keywords:** PyTorch, deep learning, primary ciliary dyskinesia, computed tomography, PCD

## Abstract

**Objective:** To analyze the cranial computed tomography (CT) imaging features of patients with primary ciliary dyskinesia (PCD) who have exudative otitis media (OME) and sinusitis using a deep learning model for early intervention in PCD.

**Methods:** Thirty-two children with PCD diagnosed at the Children’s Hospital of Fudan University, Shanghai, China, between January 2010 and January 2021 who had undergone cranial CT were retrospectively analyzed. Thirty-two children with OME and sinusitis diagnosed using cranial CT formed the control group. Multiple deep learning neural network training models based on PyTorch were built, and the optimal model was trained and selected to observe the differences between the cranial CT images of patients with PCD and those of general patients and to screen patients with PCD.

**Results:** The Swin-Transformer, ConvNeXt, and GoogLeNet training models had optimal results, with an accuracy of approximately 0.94; VGG11, VGG16, VGG19, ResNet 34, and ResNet 50, which are neural network models with fewer layers, achieved relatively strong results; and Transformer and other neural networks with more layers or neural network models with larger receptive fields exhibited a relatively weak performance. A heat map revealed the differences in the sinus, middle ear mastoid, and fourth ventricle between the patients with PCD and the control group. Transfer learning can improve the modeling effect of neural networks.

**Conclusion:** Deep learning-based CT imaging models can accurately screen for PCD and identify differences between the cranial CT images.

## 1 Introduction

Primary ciliary dyskinesia (PCD) is a genetic disorder in which the mucociliary clearance ability is impaired as a result of defects in the structure or function of cilia (Afzelius, 1976). PCD is generally believed to be an autosomal dominant disorder, but some studies have also recognized it as an X chromosome genetic disease. The prevalence rate of PCD is approximately 1/30,000–1/10,000 and is the same for male and female patients (Knowles et al., 2013).

PCD can cause symptoms in multiple systems of the body (Leigh et al., 2009). Patients with PCD can present with symptoms similar to those for respiratory diseases, such as respiratory infections, chronic bronchitis, and bronchiectasis, leading to a high rate of underdiagnosis and misdiagnosis; thus, the actual incidence of PCD may be higher than currently reported (Mirra et al., 2017). Patients with PCD also present with symptoms of otolaryngology-related diseases, such as chronic sinusitis, nasal polyps, and exudative otitis media (OME). The incidence of sinusitis in adult patients with PCD is as high as 90%, and the incidence of nasal polyps in adult patients with PCD is 33%–50% (Rollin et al., 2009). OME is a sterile accumulation of fluid in the tympanic chamber of the middle ear (Lee, 2013) that becomes viscous over time and can lead to a hearing threshold approximately 20 dB higher than is normal in children under 3 years of age (Lucas et al., 2017). The incidence of OME in patients with PCD, particularly in children, is 50%–90% (Guan et al., 2021). In addition, patients with PCD may present with central nervous system disorders, immunodeficiencies, infertility, visceral inversions, and abnormalities of other organs (Lee, 2013).

The diagnosis of PCD relies primarily on transmission electron microscopy (TEM) techniques, which demonstrate, for example, the absence of kinesin arms or radiation spokes and lack of or additional microtubule assembly. High-speed videomicroscopy analysis combined with ciliary rhythmic movement frequency testing can determine whether cilia coordination, frequency, and the pattern of rhythmic movement are normal (Lucas et al., 2017). In addition, genetic testing can confirm a PCD diagnosis, often identifying pure or compound heterozygous pathogenic variants or the co-existence of different pathogenic genotypes of different PCD genes (Guan et al., 2021).

Screening out patients with PCD from those with common otolaryngological diseases is essential. However, traditional diagnostic methods are cumbersome, time consuming, and financially costly. Radiomics was first proposed by Dutch scholars in 2012 (Lambin et al., 2017; Lambin et al., 2012), with an emphasis on obtaining high-throughput imaging information from imaging results [magnetic resonance imaging (MRI), CT, positron emission tomography, etc.] for tumor feature extraction, segmentation, and model building to assist clinicians in making more accurate diagnoses.

In 2012, computer vision counting flourished after the AlexNet network was proposed (Krizhevsky et al., 2012). In recent years, the integration of medical image-assisted diagnosis technology and big data has produced a new radiomics method (Gillies et al., 2016). As this research method has developed, an increasing number of scholars have begun using data such as imaging and computer vision to evaluate various disease phenotypes (Silva et al., 2021). However, this approach is not commonly used in otorhinolaryngology head and neck surgery. In this study, we used the imaging performance of temporal bone CT and deep learning for the first time to screen for PCD.

## 2 Materials and methods

### 2.1 General data

A retrospective analysis of the clinical data of 64 patients who attended the Ear, Nose, and Throat Department of the Children’s Hospital of Fudan University, Shanghai, China, between January 2010 and January 2021 was conducted. The study was approved by the ethics committee of the Children’s Hospital of Fudan University. The patients were divided into a PCD group and control group. The diagnosis and treatment of primary ciliary dyskinesia patients is handled by a pulmonologist (lung specialist) and otolaryngologist (ear, nose, and throat specialist). The main symptoms of primary ciliary dyskinesia (PCD) include chronic respiratory infections (such as bronchitis and pneumonia), chronic sinusitis, and abnormal mucus production in the lungs. Other symptoms may include recurrent ear infections, hearing loss, and infertility. Diagnosis of PCD is typically made through a combination of clinical examination, imaging studies, and laboratory tests such as ciliary beat frequency analysis, electron microscopy, and genetic testing.

#### 2.1.1 PCD group

The American Thoracic Society guidelines (The Lancet Respiratory Medicine, 2018), based on the Grading of Recommendations, Assessment, Development, and Evaluation methodology, considered four key diagnostic questions relating to patients with a high probability of PCD who met two of four clinical criteria: unexplained neonatal respiratory distress as a full-term infant, year-round daily cough or nasal congestion beginning before 6 months of age, and organ laterality defect. The reference standard diagnostic methods used for comparison were TEM of ciliary defects and/or identification of biallelic causative mutations. TEM identifies specific defects, such as an absence of kinesin arms or radiation spokes and lack of or additional microtubule assembly. Gene variants include mutations in the extra-axonemal kinesin arms (DNAH5, DNAH9, DNAH12, DNAI1, ARMC4, and CCDC103), the inner kinesin arm (DNALI1), and the assembly protein (DNAAF3). Thirty-two patients (7.6 ± 4.5 years) were diagnosed with PCD according to this criterion: 23 male and nine female patients, aged from 1 to 17 years. All patients were followed up for at least 3 years.

#### 2.1.2 Control group

Patients with a CT diagnosis of OME and sinusitis who had been cured without recurrence at the 1-year follow-up. Thirty-two patients (8.5 ± 3.9 years) formed the group: 23 male and nine female patients, aged from 3 to 17 years.

The study process was divided into the following five steps: 1) The collection of raw temporal bone CT data in the Digital Imaging and Communications in Medicine format; 2) Use of LabelMe software to label each layer of the CT images; Labelme is an image tagging tool developed by MIT’s Computer Science and Artificial Intelligence Laboratory (CSAIL) that allows people to create custom tagging tasks or perform image tagging (Torralba et al., 2010). 3) Building of an image database with 3,133 images for the PCD group and 3,470 images for the control group; Select head CT of otitis media patients with corresponding age and weight as the control for the case group. In this study, cases were randomly assigned to the training set and the test set, with 81% of the cases in the training set and 19% in the test set. Head CT images (2,538 images) from 26 PCD patients were used as the training set and head CT images (595 images) from six patients were used as the validation set for training in the case group. In the control group, head CT images (2,811 images) from 26 PCD patients were used as the training set and head CT images (659 images) from six patients were used as the validation set. 4) Our deep learning workstation is a Dell T7920 with two RTX 3090 GPUs. Each deep learning network requires multiple adjustments of the learning rate and multiple training sessions to find the optimal result, with at least 800 epochs per training session. 5) Classification and prediction of deep learning neural networks. The neural networks in our research are all built based on pytorch (Contributors, 2020). The process of building a neural network based on pytorch and extracting deep learning features involves several steps: 1. Importing the necessary libraries and modules, such as torch, torchvision, and numpy. 2. Defining the architecture of the neural network, such as the number of layers, the number of neurons in each layer, and the activation functions used. 3. Initializing the weights and biases of the network using random values or pre-trained values. 4. Training the network by providing it with a dataset of input and output values, and adjusting the weights and biases based on the error between the predicted and actual outputs. 5. Extracting the deep learning features by forward-propagating the input through the trained network and obtaining the output of the final layer or intermediate layers. 6. Using the extracted features for tasks such as classification, detection, or feature extraction. 7. Testing the network on new data to evaluate its performance. Python and PyTorch algorithms were used to build the deep learning neural networks, and VGG11, VGG16, VGG19, ResNet 18, ResNet 34, ResNet 50, ConvNeXt, GoogLeNet, and Swin-Transformer (Swin-T) training models were used to classify and predict a variety of networks. Neural network models were evaluated using accuracy, precision, recall, f1 score, and area under the curve (AUC). Gradient-weighted Class Activation Mapping (Grad-CAM) provided heat mapping for visual interpretation (Selvaraju et al., 2017).

### 2.2 Statistical analysis

SPSS 22.0 software was used to analyze the data. Numerical variables were represented by mean ± standard deviation, and the inter-group difference was identified using an independent samples *t*-test. The inter-group differences of measurement data, recorded as a percentage (n ^[%]^), were analyzed using a chi-square test. *p* < 0.05 was considered statistically different.

## 3 Results

### 3.1 Accuracy, precision, recall, and f1 score parameters of different training models

The results of the parameters of each training model are presented in Table 1. VGG11, VGG16, and VGG19 are common VGG training models with different depths. They all achieved accuracy and f1 score between 0.887 and 0.904, precision between 0.883 and 0.885, and recall values between 0.888 and 0.893. However, these models had more training parameters and a slower speed than other models.

**TABLE 1:** Classification matrix.

	Accuracy	Precision	Recall	F1 score
Vgg 11	0.903	0.884	0.890	0.887
Vgg 16	0.904	0.885	0.888	0.887
Vgg 19	0.903	0.883	0.893	0.887
Resnet 34	0.897	0.872	0.906	0.884
Resnet 34 pre-train	0.931	0.916	0.923	0.919
Resnet 50	0.885	0.859	0.886	0.871
Resnet 50 pre-train	0.902	0.886	0.882	0.884
Resnet 101	0.873	0.845	0.879	0.857
Resnet 101 pre-train	0.908	0.884	0.921	0.897
Googlenet	0.939	0.941	0.942	0.939
Vit	0.684	0.598	0.569	0.570
Vit pre-train	0.877	0.851	0.874	0.860
Swin-t	0.553	0.563	0.559	0.549
Swin-t pre-train	0.943	0.938	0.925	0.931
Convnext	0.772	0.803	0.78	0.769
Convnext pre-train	0.948	0.95	0.95	0.947

ResNet 34, ResNet 50, and ResNet 101 are commonly used ResNet training models. Their accuracy ranged from 0.873 to 0.931, precision from 0.845 to 0.916, recall values from 0.879 to 0.923, and f1 score from 0.857 to 0.919. The effectiveness of these models decreased as the number of layers of the neural network increased. However, the four parameters of all three neural network models improved significantly after using pretrained weights.

The GoogLeNet training model demonstrated accuracy, precision, recall value, and f1 scores of 0.939, 0.941, 0.942, and 0.939, respectively, and it had a faster training speed.

Vision Transformers (ViT), Swin-T, and ConvNeXt are the most recently developed neural network models, with accuracies between 0.684 and 0.772, precision between 0.563 and 0.803, recall between 0.549 and 0.78, and f1 score between 0.549 and 0.769. With the use of pretrained weights, the four parameters of Swin-T and ConvNeXt were significantly improved to above 0.94, but the parameters of the ViT model were not significantly improved.

### 3.2 Receiver operating characteristic curves and area under the curve values of different training models with and without pretrained weights

**FIGURE 1:**
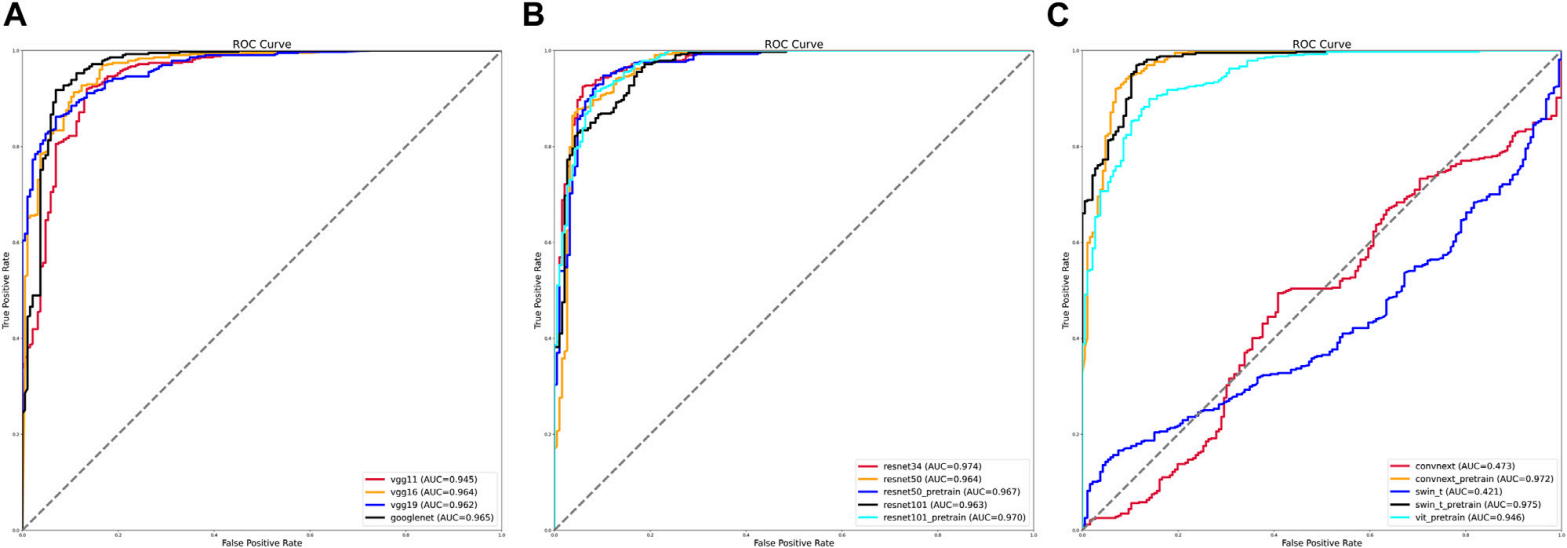
Receiver operating characteristic (ROC) curves and area under the curve (AUC) values of various training models. **(A)** ROC curves and AUC values of VGG and GoogLeNet training models. **(B)** ROC curves and AUC values of ResNet training models before and after using pretrained weights. **(C)** ROC curves and AUC values of ViT, Swin-T, and ConvNeXt training models before and after using pretrained weights.

We evaluated the various training models by calculating the AUC values under the receiver operating characteristic (ROC) curves. Of the traditional training models, the VGG11, VGG16, and VGG19 training models had fewer network layers, and their AUC values were between 0.945 and 0.962. By contrast, the curve of the GoogLeNet training model was further to the left, and its AUC value was the largest at 0.965 (Figure 1A).

The AUC values of the different training methods under the ResNet model gradually decreased as the number of network layers increased, with values ranging from 0.963 to 0.974. After using pretrained weights, the ResNet 50 training model with more layers achieved an AUC value of 0.967 (Figure 1B).

The AUC values were 0.421 for the Swin-T training model and 0.565 for the ViT training model. After using the pretrained weights, the AUC values reached 0.946 for the ViT training model, 0.975 for the Swin-T training model, and 0.972 for the ConvNeXt training model (Figure 1C).

### 3.3 Heat mapping of the middle ear mastoid, maxillary sinus, and fourth ventricle in patients with primary ciliary dyskinesia

**FIGURE 2:**
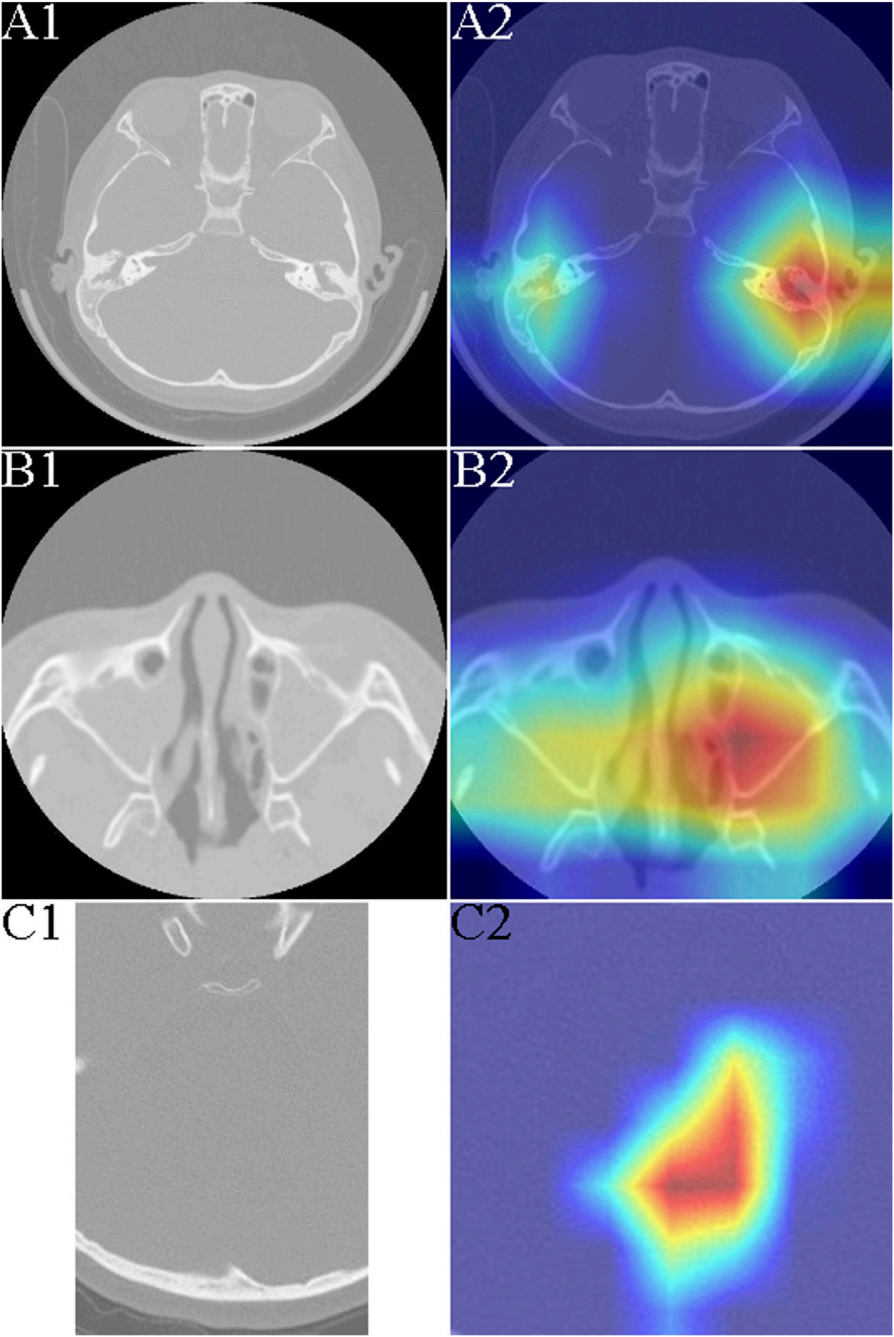
Typical heat mapping of the middle ear mastoid, maxillary sinus, and fourth ventricle in patients with primary ciliary dyskinesia (PCD) and the control group. **(A1)** Computed tomography (CT) image of middle ear mastoid in a patient with exudative otitis media (OME). **(A2)** CT image of middle ear mastoid in a patient with PCD and OME. **(B1)** CT image of maxillary sinus in a patient with sinusitis. **(B2)** CT image and heat mapping of maxillary sinus in a patient with PCD and sinusitis. **(C1)** CT image of fourth ventricle in a patient with PCD. **(C2)** CT image and heat mapping of fourth ventricle in a patient with PCD.

The otologic complications of PCD develop as a result of defective cilia function in the eustachian tube and middle ear, leading to mucus collection. Most studies have used otoscopy, audiometry, and clinical course to assess the ear features of PCD, but these methods require extensive experience and proficiency (Takeuchi et al., 2017). We found that children with PCD who have OME exhibit distinctly highlighted areas on imaging histology compared with those with common chronic OME (Figures 2A1, A2).

Frequent runny nose and lifelong nasal congestion can begin early in children with PCD (Yiallouros et al., 2015). Nasal sinusitis is the main feature of PCD and is seen in almost all patients (Shapiro et al., 2018). We did not detect nasal polyps in all patients with PCD; chronic sinusitis usually involves the maxillary and septal sinuses, but sinus CT was not significantly specific. Using the artificial intelligence (AI) model, we could see a clearly highlighted area in the maxillary sinuses bilaterally, which could help make a successful differential diagnosis of PCD (Figures 2B1, B2).

Neurologically, the symptoms of patients with PCD are mainly malaise and headache, which may be caused by chronic sinusitis, but the headache may persist even in the infection-free period. Our results revealed that the fourth ventricle was significantly different from the normal fourth ventricle even in patients with PCD without significant ventricular dilatation (Figures 2C1, C2).

## 4 Discussion

In most patients with PCD, the condition develops in childhood, with a median age of 5–5.5 years at the time of diagnosis. However, some patients develop PCD in adulthood, with a median age of 22 years at the time of diagnosis (Lucas et al., 2020). Patients with PCD often seek medical treatment for more than 50 recurrent episodes of the disease (Sommer et al., 2011), mainly because of the non-specific nature of PCD symptoms. The symptoms of PCD can be widely cumulative throughout the body and include coughing, bronchial dilatation, sinusitis, nasal polyps, otitis media, and infertility. The diagnosis of PCD is greatly hampered by the high cost of genetic testing, long reporting period, questionable nature of some genetic variants, and the invasiveness of electron microscopy. Currently, limited guidance exists on which patients should be referred for appropriate specialty testing, especially in the pediatric population. More effective methods for the diagnosis of PCD are urgently required. Tumor-related AI technologies and radiomics have developed rapidly in recent years (Guo et al., 2016) and can even indirectly reflect changes in genes or proteins at the tissue microscopic level (Castaldo et al., 2021). In this study, we first built a neural network deep learning model using PyTorch to differentially diagnose OME or sinusitis caused by PCD through cranial CT, which is expected to provide a reference for early clinical intervention in PCD.

The VGG model was proposed by the Visual Geometry Group in the Department of Science and Engineering, University of Oxford, in 2014 (Simonyan and Zisserman, 2015), and it has numerous versions because of the different depths of the network. Compared with other networks, VGG uses smaller convolutional kernels with deeper networks to improve parameter efficiency and increase the non-linear and generalization performance of the network. The drawback of the VGG model lies in the number of parameters, and VGG19 is the convolutional network architecture with the largest number of parameters. The VGG network model used in this study works better, with an accuracy above 0.93 in all cases.

ResNet, proposed by He et al. (2016) at Microsoft Research, is a classic neural network that serves as the backbone of many computer vision tasks. ResNet successfully solves the problem of vanishing gradients, thus enabling the neural network depth to exceed 100 layers. However, this network may be less suitable for the images with small target positions used in this study. The accuracy of the ResNet 101 model in this study was 0.873, which was the poorest result. This may be because the neural network has too many layers and the perceptual field is too large, which influences the model effect. However, after using pretrained weights, the results of all models were significantly improved.

GoogLeNet is a new deep learning structure proposed by Szegedy in 2014 (Szegedy et al., 2015). It is designed to improve the network performance by increasing the width of the network using convolutional kernels of different sizes in each module, which are then concentrated, enriching the information in each layer; a Bayesian network algorithm is then used to speed up the convergence of the network. Furthermore, GoogLeNet incorporates the idea of residuals from ResNet to deepen the network. In this study, the neural network model trained by GoogLeNet achieved optimal results, and its accuracy, precision, recall value, and f1 score all reached approximately 0.94.

Transformer (Vaswani et al., 2017) was proposed by Bengio’s team in 2014 and is widely used in various areas of deep learning. Its disadvantage is that it does not have the ability to capture local features and is therefore not effective for use in studies with small target images and small amounts of data. In this study, even with pretrained weights, the accuracy, precision, recall value, and f1 score of ViT was approximately 0.57. Swin-T is a new architecture proposed on this basis (Liu et al., 2021), which incorporates the convolutional neural network (CNN) and solves the disadvantages of Transformer, making it suitable for studies with small target images and small amounts of data. In this study, the accuracy, precision, recall value, and f1 score all reached above 0.94 after using pretrained weights.

ConvNeXt can be seen as a convolutional network evolution that combines the special design features of Swin-T and ViT (Liu et al., 2022). It upgrades the ResNet architecture, learning the self-attention layer of Swin-T and its architecture. ConvNeXt is more valuable in industrial deployments and clinical applications. Its accuracy, precision, recall value, and f1 score in this study were all above 0.94. ViT, Swin-T, and ConvNeXt are currently the most popular nets, but they all require large amounts of data and computing power for training. Without using their corresponding pretrained weights for transfer learning, the models in this study achieved poor results. In this study, ViT, Swin-T, and ConvNeXt achieved strong results using their corresponding pretrained weights.

Without using pretrained weights, the traditional CNN network is more suitable for medical image research with insufficient research data. In this study, with the use of pretrained weights, each net model achieved positive model results except Transformer. Users must focus on which method to use for transfer learning and identifying when migration is effective and when has negative effects. However, no methods exist to support effective migration.

The reliability of deep learning image classification can be defined as the ability of the model to consistently and accurately classify images. This can be measured by testing the model on a large dataset of labeled images and comparing the results to the true labels. The accuracy, precision, and recall of the model can also be used to determine its reliability. Primary ciliary dyskinesia is a rare disease with strict diagnostic criteria that require genetic diagnosis. Therefore, it is difficult to collect a large number of CT images, especially for multi-center studies with large sample sizes. In the deep learning networks, the final fully connected layer uses a softmax activation function for binary classification. The softmax function converts the output of the final layer into a probability distribution, where the probability of each class is represented by a value between 0 and 1 (Kouretas and Paliouras, 2019). The class with the highest probability is then chosen as the final prediction. This is known as one-hot encoding, where the predicted class is represented by a vector of all zeros with a 1 in the position of the predicted class.

Deep learning is a powerful method for solving complex problems, but it has a major limitation: it is not very interpretable. This means that it can be difficult to understand why a deep learning model is making a particular prediction. One way to improve the interpretability of deep learning models is through the use of visualization techniques such as Grad-CAM. Grad-CAM is a method that allows us to understand which parts of an image are most important for a deep learning model’s prediction. The basic process of Grad-CAM is as follows: first, the model is trained on a dataset. Next, the model is used to classify an image and the output of the final convolutional layer is obtained. The output is then passed through a global average pooling layer to obtain a feature map. This feature map is then multiplied by the gradient of the output of the final convolutional layer with respect to the input image. The resulting heatmap is then overlayed on the original image to highlight the regions that are most important for the model’s prediction.

To better understand the neural network and allow the model to make decisions, Grad-CAM was proposed to discriminate the image position without attention. In contrast to CAM, Grad-CAM can visualize CNNs of arbitrary structure without modifying the network structure or retraining. Gradient information from the last convolutional layer flowing into the CNN is used by Grad-CAM to assign importance values to each neuron for a specific attention decision. Although the technique is rather general because it can be used to interpret activations in any layer of the deep network, this study focused only on interpreting decisions in the output layer.

The targets of this study are the middle ear tympanic chambers, sinuses, and ventricles, which are areas of high secretion and have a small proportion of area monolayer image pictures. Classifying images generally requires up to a million images for effective modeling. However, collecting so much data in medical research is challenging, especially for rare diseases. Transfer learning is a machine learning method that can migrate the trained model parameters to a new model to help new model training (Niu et al., 2021). If the training data are small and the training model of deep learning is less effective, transfer learning can be considered.

This study has some limitations. Primary ciliary dyskinesia is an extremely rare disease. Collecting cases is extremely difficult. For fine-grained image classification, the minimum number of images required will depend on the specific task and dataset. However, it is generally recommended to have a minimum of several hundred images per class for a fine-grained classification task (Shahinfar et al., 2020). Due to the small number of cases, this study only divides into training and testing sets and does not have a validation set. This limitation is mentioned in this paper. We will conduct multi-center research and collect more cases, using external datasets to validate. The small number of cases in this study and the insufficient follow-up time of the patients do not allow us to confirm the resolution of OME, sinusitis, and other organ abnormalities in the participating children. In addition, this study is a single-center study, with no other data available for validation. In the future, we will combine genetic testing, CT, and other sequences of MRI for multimodal studies, as well as the three-dimensional (3D) separation of the middle ear mastoid, Eustachian tube, and surrounding tissues for 3D model studies.

In conclusion, our deep learning-based imaging histological characterization is a vital aid in the differential diagnosis of OME and PCD and provides important clues for early non-invasive diagnosis.

## Data Availability

The original contributions presented in the study are included in the article/supplementary material, further inquiries can be directed to the corresponding authors.

## References

[B1] Afzelius B. A. (1976). A human syndrome caused by immotile cilia. Science 193 (4250), 317–319. 10.1126/science.1084576 1084576

[B2] Castaldo R. Cavaliere C. Soricelli A. Salvatore M. Pecchia L. Franzese M. (2021). Radiomic and genomic machine learning method performance for prostate cancer diagnosis: Systematic literature review. J. Med. Internet Res. 23 (4), e22394. 10.2196/22394 33792552 PMC8050752

[B3] Contributors M. (2020). OpenMMLab's image classification toolbox and benchmark. Available at: https://github.com/open-mmlab/mmclassification .

[B4] Gillies R. J. Kinahan P. E. Hricak H. (2016). Radiomics: Images are more than pictures, they are data. Radiology 278 (2), 563–577. 10.1148/radiol.2015151169 26579733 PMC4734157

[B5] Guan Y. Yang H. Yao X. Xu H. Liu H. Tang X. (2021). Clinical and genetic spectrum of children with primary ciliary dyskinesia in China. Chest 159 (5), 1768–1781. 10.1016/j.chest.2021.02.006 33577779 PMC8129725

[B6] Guo Y. Gao Y. Shen D. (2016). Deformable MR prostate segmentation via deep feature learning and sparse patch matching. IEEE Trans. Med. Imaging 35 (4), 1077–1089. 10.1109/TMI.2015.2508280 26685226 PMC5002995

[B7] He K. Zhang X. Ren S. Sun J. (2016). “Deep residual learning for image recognition,” in 2016 IEEE Conference on Computer Vision and Pattern Recognition (CVPR), Las Vegas, NV, USA, 27-30 June 2016, 770–778.

[B8] Knowles M. R. Daniels L. A. Davis S. D. Zariwala M. A. Leigh M. W. (2013). Primary ciliary dyskinesia. Recent advances in diagnostics, genetics, and characterization of clinical disease. Am. J. Respir. Crit. Care Med. 188 (8), 913–922. 10.1164/rccm.201301-0059CI 23796196 PMC3826280

[B9] Kouretas I. Paliouras V. (2019). “Simplified hardware implementation of the softmax activation function,” in 2019 8th International Conference on Modern Circuits and Systems Technologies (MOCAST), Thessaloniki, Greece, 13-15 May 2019.

[B10] Krizhevsky A. Sutskever I. Hinton G. E. (2012). Imagenet classification with deep convolutional neural networks. Adv. neural Inf. Process. Syst. 25.

[B11] Lambin P. Leijenaar R. T. H. Deist T. M. Peerlings J. de Jong E. E. C. van Timmeren J. (2017). Radiomics: The bridge between medical imaging and personalized medicine. Nat. Rev. Clin. Oncol. 14 (12), 749–762. 10.1038/nrclinonc.2017.141 28975929

[B12] Lambin P. Rios-Velazquez E. Leijenaar R. Carvalho S. van Stiphout R. G. P. M. Granton P. (2012). Radiomics: Extracting more information from medical images using advanced feature analysis. Eur. J. Cancer 48 (4), 441–446. 10.1016/j.ejca.2011.11.036 22257792 PMC4533986

[B13] Lee L. (2013). Riding the wave of ependymal cilia: Genetic susceptibility to hydrocephalus in primary ciliary dyskinesia. J. Neurosci. Res. 91 (9), 1117–1132. 10.1002/jnr.23238 23686703

[B14] Leigh M. W. Pittman J. E. Carson J. L. Ferkol T. W. Dell S. D. Davis S. D. (2009). Clinical and genetic aspects of primary ciliary dyskinesia/Kartagener syndrome. Genet. Med. 11 (7), 473–487. 10.1097/GIM.0b013e3181a53562 19606528 PMC3739704

[B15] Liu Z. Lin Y. Cao Y. Hu H. Wei Y. Zhang Z. (2021). “Swin transformer: Hierarchical vision transformer using shifted windows,” in 2021 IEEE/CVF International Conference on Computer Vision (ICCV), Montreal, QC, Canada, 10-17 October 2021.

[B16] Liu Z. Mao H. Wu C.-Y. Feichtenhofer C. Darrell T. Xie S. (2022). A ConvNet for the 2020s. arXiv preprint arXiv:2201.03545.

[B17] Lucas J. S. Barbato A. Collins S. A. Goutaki M. Behan L. Caudri D. (2017). European Respiratory Society guidelines for the diagnosis of primary ciliary dyskinesia. Eur. Respir. J. 49 (1), 1601090. 10.1183/13993003.01090-2016 27836958 PMC6054534

[B18] Lucas J. S. Davis S. D. Omran H. Shoemark A. (2020). Primary ciliary dyskinesia in the genomics age. Lancet Respir. Med. 8 (2), 202–216. 10.1016/S2213-2600(19)30374-1 31624012

[B19] Mirra V. Werner C. Santamaria F. (2017). Primary ciliary dyskinesia: An update on clinical aspects, genetics, diagnosis, and future treatment strategies. Front. Pediatr. 5, 135. 10.3389/fped.2017.00135 28649564 PMC5465251

[B20] Niu S. Liu M. Liu Y. Wang J. Song H. (2021). Distant domain transfer learning for medical imaging. IEEE J. Biomed. Health Inf. 25 (10), 3784–3793. 10.1109/JBHI.2021.3051470 PMC8545174 33449887

[B21] Rollin M. Seymour K. Hariri M. Harcourt J. (2009). Rhinosinusitis, symptomatology & absence of polyposis in children with primary ciliary dyskinesia. Rhinology 47 (1), 75–78. 19382500

[B22] Selvaraju R. R. Cogswell M. Das A. Vedantam R. Parikh D. Batra D. (2017). “Grad-cam: Visual explanations from deep networks via gradient-based localization,” in 2017 IEEE International Conference on Computer Vision (ICCV), Venice, Italy, 22-29 October 2017.

[B23] Shahinfar S. Meek P. D. Falzon G. (2020). How many images do I need?" Understanding how sample size per class affects deep learning model performance metrics for balanced designs in autonomous wildlife monitoring. Ecol. Inf. 57, 101085. 10.1016/j.ecoinf.2020.101085

[B24] Shapiro A. J. Davis S. D. Polineni D. Manion M. Rosenfeld M. Dell S. D. (2018). Diagnosis of primary ciliary dyskinesia. An official American thoracic society clinical practice guideline. Am. J. Respir. Crit. Care Med. 197 (12), e24–e39. 10.1164/rccm.201805-0819ST 29905515 PMC6006411

[B25] Silva N. Zhang D. Kulvicius T. Gail A. Barreiros C. Lindstaedt S. (2021). The future of General Movement Assessment: The role of computer vision and machine learning - a scoping review. Res. Dev. Disabil. 110, 103854. 10.1016/j.ridd.2021.103854 33571849 PMC7910279

[B26] Simonyan K. Zisserman A. (2015). Very deep convolutional networks for large-scale image recognition. arXiv:1409.1556v6.

[B27] Sommer J. U. Schafer K. Omran H. Olbrich H. Wallmeier J. Blum A. (2011). ENT manifestations in patients with primary ciliary dyskinesia: Prevalence and significance of otorhinolaryngologic co-morbidities. Eur. Arch. Otorhinolaryngol. 268 (3), 383–388. 10.1007/s00405-010-1341-9 20652291

[B28] Szegedy C. Liu W. Jia Y. Sermanet P. Reed S. Anguelov D. (2015). “Going deeper with convolutions,” in 2015 IEEE Conference on Computer Vision and Pattern Recognition (CVPR), Boston, MA, USA, 07-12 June 2015, 1–9.

[B29] Takeuchi K. Kitano M. Sakaida H. Usui S. Masuda S. Ogawa S. (2017). Analysis of otologic features of patients with primary ciliary dyskinesia. Otol. Neurotol. 38 (10), e451–e456. 10.1097/MAO.0000000000001599 29135867

[B30] The Lancet Respiratory Medicine (2018). Guideline provides new diagnostic insights for PCD. Lancet Respir. Med. 6 (8), 567. 10.1016/S2213-2600(18)30304-7 30070255

[B31] Torralba A. Russell B. Yuen J. (2010). LabelMe: Online image annotation and applications. Proc. IEEE 98, 1467–1484. 10.1109/jproc.2010.2050290

[B32] Vaswani A. Shazeer N. Parmar N. Uszkoreit J. Jones L. Aidan N. (2017). “Attention is all you need,” in Advances in neural information processing systems. 30.

[B33] Yiallouros P. K. Kouis P. Middleton N. Nearchou M. Adamidi T. Georgiou A. (2015). Clinical features of primary ciliary dyskinesia in Cyprus with emphasis on lobectomized patients. Respir. Med. 109 (3), 347–356. 10.1016/j.rmed.2015.01.015 25698650

